# The impact of HLA-Cw*12:02 on control of HIV-1 infection

**DOI:** 10.1186/1742-4690-9-S2-P257

**Published:** 2012-09-13

**Authors:** M Koyanagi, K Honda, T Chikata, T Akahoshi, H Murakoshi, H Gatanaga, S Oka, M Takiguchi

**Affiliations:** 1 Center for AIDS Research, Kumamoto University, Kumamoto, Japan; 2 AIDS Clinical Center, National Center for Global Health and Medicine, Tokyo, Japan

## Background

Previous studies have demonstrated that higher HLA-C expression, which is determined through a single nucleotide polymorphism 35 kb upstream and the variation within the 3’ untranslated region of the HLA-C locus, associate with slow progression of HIV-1-infected disease. Although HLA-C plays important roles in presenting antigens to CTLs or a ligand for inhibitory killer cell Ig-like receptors (KIR), the role of HLA-C-restricted CTL and NK cells in the control of HIV-1 is still unclear. Our recent study of chronically HIV-1 infected Japanese cohort showed that the HLA-B*52:01-Cw*12:02 haplotype was significantly associated with lower viral load. In this study, we investigated whether HLA-Cw*12:02-restricted CTLs or NK cells via KIR have a significant impact on viraemic control.

## Methods

We sequenced Pol, Gag and Nef from 400 chronically HIV-1 clade B-infected treatment-naïve Japanese individuals and then analyzed amino acid polymorphisms associated with HLA-B*52:01-Cw*12:02 haplotype using Fisher’s exact test. Next we performed intracellular IFNg staining or IFNg ELISPOT assay to detect CTL responses to the peptides including those polymorphisms.

## Results

We found 9 amino acid polymorphisms significantly associated with HLA-B*52:01-Cw*12:02 haplotype (p<0.002 q<0.2). By using ICC assay, we identified 2 Cw*12:02-restricted CTL epitopes and 4 B*52:01-restricted ones. Four Cw*12:02-restricted CTL epitopes including previously reported ones were analyzed to investigate the effect of Cw*12:02-restricted CTLs on control of HIV-1. No significant correlation between the responses to these Cw*12:02-restricted epitopes and viral load was found in chronically HIV-1 infected Cw*12:02 positive Japanese individuals.

## Conclusion

Those results showed that HLA-Cw*12:02-restrected CTLs have no effect on control of HIV-1 and suggested that HLA-B*52:01-restrected CTLs or Cw*12:02-resticted NK cells control HIV-1 viraemia in Japanese cohort.

